# Pyoverdine synthesis by the Mn(II)-oxidizing bacterium *Pseudomonas putida* GB-1

**DOI:** 10.3389/fmicb.2014.00202

**Published:** 2014-05-07

**Authors:** Dorothy L. Parker, Sung-Woo Lee, Kati Geszvain, Richard E. Davis, Christelle Gruffaz, Jean-Marie Meyer, Justin W. Torpey, Bradley M. Tebo

**Affiliations:** ^1^Geosciences Research Division, Scripps Institution of Oceanography, University of California San DiegoLa Jolla, CA, USA; ^2^Division of Environmental and Biomolecular Systems, Oregon Health and Science UniversityBeaverton, OR, USA; ^3^Laboratoire de Génétique Moléculaire, Génomique et Microbiologie, Université de StrasbourgStrasbourg, France; ^4^Biomolecular Mass Spectrometry Facility, Department of Chemistry and Biochemistry, University of California San DiegoLa Jolla, CA, USA

**Keywords:** siderophore, pyoverdine, azotobactin, manganese oxidation, iron

## Abstract

When iron-starved, the Mn(II)-oxidizing bacteria *Pseudomonas putida* strains GB-1 and MnB1 produce pyoverdines (PVD_GB-1_ and PVD_MnB1_), siderophores that both influence iron uptake and inhibit manganese(II) oxidation by these strains. To explore the properties and genetics of a PVD that can affect manganese oxidation, LC-MS/MS, and various siderotyping techniques were used to identify the peptides of PVD_GB-1_ and PVD_MnB1_ as being (for both PVDs): chromophore-Asp-Lys-OHAsp-Ser-Gly-aThr-Lys-cOHOrn, resembling a structure previously reported for *P. putida* CFML 90-51, which does not oxidize Mn. All three strains also produced an azotobactin and a sulfonated PVD, each with the peptide sequence above, but with unknown regulatory or metabolic effects. Bioinformatic analysis of the sequenced genome of *P. putida* GB-1 suggested that a particular non-ribosomal peptide synthetase (NRPS), coded by the operon PputGB1_4083-4086, could produce the peptide backbone of PVD_GB-1_. To verify this prediction, plasmid integration disruption of PputGB1_4083 was performed and the resulting mutant failed to produce detectable PVD. *In silico* analysis of the modules in PputGB1_4083-4086 predicted a peptide sequence of Asp-Lys-Asp-Ser-Ala-Thr-Lsy-Orn, which closely matches the peptide determined by MS/MS. To extend these studies to other organisms, various Mn(II)-oxidizing and non-oxidizing isolates of *P. putida, P. fluorescens, P. marincola, P. fluorescens-syringae* group, *P. mendocina-resinovorans* group, and *P. stutzerii* group were screened for PVD synthesis. The PVD producers (12 out of 16 tested strains) were siderotyped and placed into four sets of differing PVD structures, some corresponding to previously characterized PVDs and some to novel PVDs. These results combined with previous studies suggested that the presence of OHAsp or the flexibility of the pyoverdine polypeptide may enable efficient binding of Mn(III).

## Introduction

The global manganese oxidation-reduction cycle, which depends on microbial activities that increase the manganese oxidation rate by up to 5 orders of magnitude (Hastings and Emerson, 1986; Tebo et al., 2004), strongly influences the cycling of organic compounds, pollutants, and many elements including carbon, arsenic, uranium, and chromium (Tebo et al., 2004). Among the most prevalent Mn(II)-oxidizing bacteria are various *Pseudomonas* species, which oxidize soluble Mn^2+^ to insoluble Mn(IV)oxides that accumulate in late logarithmic and early stationary growth phases (Toner et al., 2005). These Mn oxides coat the cells with dark brown precipitates of nanoparticulate MnO_2_, birnessite-type minerals that exhibit large surface areas and efficient adsorption of toxic metals and organics (Villalobos et al., 2003, 2006), contributing to the environmental importance of this process. Oxidation of Mn^2+^ by all tested pseudomonads is enzymatic and utilizes oxygen as an electron acceptor (Okazaki et al., 1997; Brouwers et al., 1999; Francis and Tebo, 2001).

The model Mn(II)-oxidizing *Pseudomonas putida* strains GB-1 and MnB1 are typical representatives of the widely-distributed and diverse group of several “fluorescent *Pseudomona*s” species, which synthesize fluorescent iron-chelating compounds (siderophores) called pyoverdines (PVDs) to scavenge iron in iron-starved conditions (Budzikiewicz, 1993; Albrecht-Gary et al., 1994; Schalk et al., 2002). However, PVDs also form strong complexes with Mn(III) and can inhibit the enzymatic formation of MnO_2_ by *P. putida* GB-1 and MnB1 (Parker et al., 2004, 2007), raising several interesting questions about the interplay of iron and manganese metabolisms in these organisms and in the environment.

The PVDs of various fluorescent pseudomonads share the same chromofluorophore [(1S)-5-amino-2,3-dihydro-8,9-dihydroxy-1*H*-pyrimido-[1,2-a]quinoline-1-carboxylic acid], but can differ in an attached peptide chain that is recognized by a strain-specific PVD uptake receptor on the cell surface (Fuchs et al., 2001; Clement et al., 2004; Schons et al., 2005, Shen et al., 2005). Most isolates synthesize and recognize a suite of PVDs, usually with the same peptide but with various modifications including the addition of acyl chains to the chromophore, sulfonation of the chromophore, or the formation of azotobactin, in which an extra 5-membered ring is added to the PVD chromophore (Fuchs et al., 2001). Both the peptide that comprises the backbone of a PVD and the chromophore are synthesized by non-ribosomal peptide synthetases (NRPSs) (Ravel and Cornelis, 2003). NRPSs are large enzymes containing multiple modules; the number and order of these modules generally correlate to the number and order of (modified or unmodified) amino acids in the peptides (Ravel and Cornelis, 2003). Among the domains found within each of the modules, the adenylation domains specifically recognize and activate corresponding amino acids. This property enables *in silico* predictions concerning the amino acid sequence corresponding to a particular NRPS (Rausch et al., 2005). The sequence of the peptide backbone of a PVD can also be indirectly achieved by siderotyping. Siderotyping compares unknown PVDs with standard PVDs in terms of isoelectric focusing, microbiological uptake studies with ^59^Fe-PVD standards of differing structures (which defines the specificity of a strain's PVD uptake receptor) and, if necessary, mass spectrometric (MS) techniques adapted to PVDs (Fuchs and Budzikiewicz, 2001; Fuchs et al., 2001; Meyer et al., 2008). Among other things, siderotyping rapidly screens whether a strain produces a previously-described or a novel PVD and can aid in determining the sequence of the peptide backbone, confirmed by MS/MS.

The present study aims: (1) to identify NRPSs responsible for synthesis of the peptide backbone of the PVD produced by *P. putida* GB-1, using genomic and genetic analyses; (2) to describe the structure of this PVD based on *in silico* predictions coupled with siderotyping and MS/MS determinations; and (3) assess whether there is any correlation between PVD structure or siderotype and the ability to oxidize Mn(II). Additionally, siderotyping of the PVDs synthesized by several other Mn(II)-oxidizing *Pseudomonas* species from diverse environments was also included to define a set of PVDs with varying peptide composition and differing uptake receptor specificity for use in future investigations of PVD effects on Mn(II) oxidation in pseudomonads.

## Materials and methods

### *in silico* analysis

For phylogenetic analysis, a maximum likelihood tree was constructed with NRPS sequences of known capabilities. Sequences used include the PvdI, J and D proteins of *P. aeruginosa* PAO1 (PA2399-2402), Psyr_1957-1960 from *P. syringae* pv. *syringae* B728a (Ravel and Cornelis, 2003), and the SypC protein of *P. syringae* pv. *syringae* B301D (Scholz-Schroeder et al., 2003). The adenylation modules from each protein were identified by the NRPSpredictor (http://www-ab.informatik.uni-tuebingen.de/software/NRPSpredictor) (Rausch et al., 2005), which was also used to predict the identity of the amino acid incorporated into the peptide by each domain, for a total of 35 modules of ~150 amino acids, including the 8 modules from *P. putida* GB-1. Translated sequences were aligned using the MUSCLE multiple alignment program (Edgar, 2004) using the default parameters. A maximum likelihood phylogenetic tree was then calculated with the PROML program in the PHYLIP package (Felsenstein, 2005) using the Jones-Taylor-Thornton probability model (Jones et al., 1994). One hundred bootstrap replicates were calculated using these same conditions.

### Mass spectrometric analysis

For mass spectrometric determinations, 72-h cultures (3–4 L) of *P. putida* CFML 90-51 (Sultana et al., 2000), *P. putida* MnB1(Caspi et al., 1998), and *P. putida* GB-1 (Corstjens et al., 1992), which had been grown at 20–25^°^C with shaking in low-iron casamino acids medium (Meyer et al., 1997), were centrifuged, filtered, and adjusted to pH 5.5 with HNO_3_. Each filtrate was adsorbed to a 35 cc SepPak C18 column, washed with 5 volumes of Milli-Q deionized water (18.2 MΩ), eluted with 50% methanol in deionized water, evaporated to dryness in a SpeedVac freeze-drier (Savant Corp.), and resuspended to 2–4 mM PVD in MilliQ water. The PVDs from strains CFML 90-51 and GB-1 were each diluted 10,000-fold with MilliQ water and mixed 50:50 with α-cyano-4-hydroxycinnamic acid (Agilent G2037A). Each mixture was spotted (1 μ L) on a stainless steel MALDI plate and analyzed on a 4800 MALDI-TOF/TOF mass spectrometer (Applied Biosystems) in reflector positive mode. Tandem mass spectrometry was acquired using 2 kV collision energy with collision-induced dissociation. The PVD of strain MnB1, which was analyzed at a different time, was diluted 1000-fold using 50% acetonitrile in 0.1% formic acid and analyzed by electrospray ionization on a QSTAR hybrid QqTOF mass spectrometer (Applied Biosystems) infused at 10 μL·min^−1^ in positive mode.

### Plasmid integration into PputGB1_4083

Strains and plasmids used are summarized in Table 1. To generate a plasmid integration disruption mutation of PputGB1_4083 (PputGB1_4083::pKG220), a homolog of *pvdI*, an ~1 kb region within the gene was amplified using primer fliF_2-R (ACGATGTCCAGGCGCACC). This primer was fortuitously discovered to anneal near the 3' end of PputGB1_4083 on opposite strands of the DNA, producing a 1 kb product. The PCR product was first cloned into the specialized PCR cloning plasmid pJET1.2/blunt (Fermentas) then subcloned into pKG161, a derivative of pEX18Gm (Table 1) from which the *sacB* gene had been deleted by digestion with MscI/SnaBI and self-ligation of the plasmid backbone. This plasmid (pKG220) was moved by conjugation into *P. putida* GB-1 (Geszvain and Tebo, 2010) and transconjugants were screened for Gm resistance. Gm^R^ colonies were screened for homologous recombination between the plasmid and the chromosome, resulting in integration of the plasmid into the chromosome, by isolating genomic DNA from candidate colonies and screening by PCR using the M13-F primer, which anneals within the plasmid, and the 4083_1-F primer (GGGCCGACCATCAGGTGAAAG), which anneals within PputGB1_4083 immediately upstream of the region present on pKG220. The ability to amplify an ~1 kb product with these primers indicated that the plasmid had integrated into the chromosome to generate PputGB1_4083::pKG220.

**Table 1:** **Bacterial strains and plasmids used in genetic studies in this work**.

	**Characteristics**	**References**
***P. putida* GB-1 STRAINS**		
GB-1	Wild type	Corstjens et al., 1992
KG163	PputGB1_4083::pKG220, Gm^R^	This work
KG165	*glmS*::pKG222, Gm^R^	This work
**PLASMIDS**		
pEX18Gm	Gene replacement vector, Gm^R^, *oriT, sacB*	Hoang et al., 1998
pJET1.2/blunt	Commercial cloning vector	Fermentas
pKG161	pEX18Gm with MscI/SnaBI fragment removed	This work
pKG220	pKG161 with ~1 kb internal fragment from PputGB1_4083 cloned into the BamHI site	This work
pKG222	pKG161 with ~300 bp from the *att*Tn7 region cloned into the BamHI site	This work

To address the concern that the presence of the plasmid backbone itself could affect the phenotype of the bacteria, we also generated a plasmid integration strain in which the plasmid was inserted downstream from PputGB1_5427 (*glmS*) in the *att*Tn7 region of the chromosome. This region commonly tolerates insertions without affecting cell growth/behavior (Choi et al., 2005). The *att*Tn7 region was amplified using primers glmS-F (GTTGGTTGTGTTCGCCGACG) and glmS-R (TTCAAGGCAGCGGAGGGG), and then cloned into pKG161 to generate plasmid pKG222 as described above. Plasmid integrants were generated as above and screened via PCR with the M13-F primer and the *att*Tn7 region primer glmS_3-F (GCGCCGAACAACGAACTGC).

### Test of NRPS mutant

The wild-type equivalent, *glmS*::pKG222, and NRPS mutant, *pvdI*::pKG220, were grown in LB containing 50 μg ml^−1^ gentamicin and streaked out on LB plates supplemented with 36 μM FeSO_4_ with either 100 μM or 1 mM 2'-2' dipyridyl (Lehoux et al., 2000), CAS-CAA (Matthijs et al., 2004), or succinate medium (Meyer et al., 1997) to determine production of PVDs or siderophores.

### Comparison of mn-oxidizing strains

Table 2 lists the strains examined. For tests of siderophore production, each organism was serially transferred three times in each of two media: low-iron casamino acids medium (Meyer et al., 1997) and succinate minimal medium without added iron (Meyer et al., 1997). Chrom azurol S (CAS) tests of general siderophore presence in centrifuged and filtered culture supernatants were performed by the standard shuttle method (Schwyn and Neilands, 1987). For PVD detection, culture supernatants (ca. pH 7.7) were adjusted to pH 5, 6, or 8 with HCl or NaOH, transferred to quartz cuvettes (1 cm path length) and examined in a SpectraMax M2 scanning spectrophotometer-fluorimeter (Molecular Devices). PVD was identified by its known fluorescence (excitation at 405 nm; emission read at 470 and 535 nm, pH 8) and by its characteristic UV-vis absorbance properties as a function of pH (Albrecht-Gary et al., 1994; Parker et al., 2004, 2007), with screening for absorbance maxima at ca. 364 (pH 5), 380 (pH 6), and 400–405 nm (pH 7.5–8). Isoelectric focusing and biological uptake of ^59^Fe-PVD standards were as previously described (Fuchs et al., 2001; Meyer et al., 2007), with PVD standards from: *Pseudomonas putida* CFML 90-44, *Pseudomonas* sp. G76, *Pseudomonas* sp. G4, *P. putida* CFML 90-51, *P. putida* GS43, *P. costantinii* CFBP 5705_T_, *P. fluorescens* W, *P. monteilii* CFML 90-54, *P. putida* GS37, *P. aeruginosa* Pa6, *Pseudomonas* sp. 2908, *P. putida* WCS358, *P. fluorescens* PL7, *Pseudomonas* sp. B10, *P. fluorescens* 51W, *P. fluorescens* Pflii, *Pseudomonas* sp. CFML 96-188, *P. fluorescens* Pfl12, *Pseudomonas* sp. D47, *P. thivervalensis* ML45, *P. fluorescens* Pf0-1, *P. putida* AP3, *Pseudomonas* sp. G85, *Pseudomonas* sp. F317, and *Pseudomonas* sp. F360 (abbreviations: CFML, Collection de la Faculté de Médecine de Lille, France; CFBP, Collection Française de Bactéries Phytopathogènes, Angers, France).

**Table 2:** **Properties of various *Pseudomonas* sp. strains examined**.

**Strain**	***Pseudomonas* species or group (gp), from 16S rRNA^a^**	**Forms MnO_2_**	**CAS reaction^b^ (PVD reaction)^b^**	**Sidero-type^c^**	**^59^Fe-PVD uptake (% of homologous uptake)^d^**	**Isolated from^e^ (references in footnote)**
CFML 90-45	*putida*	No	CAS+ (PVD+)	1	CFML 90-51 (>90%)	Clinical specimen
CFML 90-48	*putida*	No	CAS+ (PVD+)	1	CFML 90-51 (>90%)	Clinical specimen
CFML 90-49	*putida*	No	CAS+ (PVD+)	1	CFML 90-51 (>90%)	Clinical specimen
CFML 90-50	*putida*	No	CAS+ (PVD+)	1	CFML 90-51 (>90%)	Clinical specimen
CFML 90-51	*putida*	No	CAS+ (PVD+)	1	CFML 90-51 (100%)	Clinical specimen
GB-1	*putida*	Yes	CAS+ (PVD+)	1	CFML 90-51 (95%)	Freshwater sediment
MnB1	*putida*	Yes	CAS+ (PVD+)	1	CFML 90-51 (104%)	Freshwater pipe
KT2440	*putida*	Yes, at low O_2_	CAS+ (PVD+)	2	F317 (91%)	Soil, toluate deg
ATCC 55241	*fluorescens* biotype II BNL-WVC	No	CAS+ (PVD+)	3	No match to known PVD	Radiowaste leachate
ISO6	*fluorescens-syringae* gp.	Yes, at low O_2_	CAS+ (PVD+)	4		Metallogenium particles
PCP1	*fluorescens-syringae* gp.	Yes	CAS+ (PVD+)	4	D47, SB8.3 (~50% each)	Sediment, mine drainage
MG1	*fluorescens-syringae* gp.	Yes	CAS+ (PVD+)	NT^f^		Metallogenium particles
ISO1	*fluorescens-syringae* gp.	Yes	CAS+ (PVD−)	NA^g^		Metallogenium particles
GP11	*stutzeri* gp.	Yes	CAS− (PVD−)	NA		Pulpmill effluent
SI85-2B	*marincola*	Yes	CAS^NT^ (PVD−)	NA		Marine bay, suboxic
PCP2	*mendocina-resinovorans* gp.	Yes	CAS− (PVD−)	NA		Sediment, mine effluent

a Based on 16S rRNA sequence (Francis and Dodge, 1998; Francis and Tebo, 2001; Meyer et al., 2007).

b The chrom azurol S (CAS) method, which depends on the ability of siderophores to displace Fe from its CAS complex, is a general assay for siderophores (Schwyn and Neilands, 1987). The presence of pyoverdine-group siderophores (PVD) was detected from UV-vis absorption and fluorescence spectra (Parker et al., 2007). A strain was scored positive (+) if a CAS reaction or PVD was detected.

c Based on the isolectric focusing pattern of each strain's fluorescent PVD and confirmed by each strain's uptake of ^59^Fe-PVD from 34 standard strains, using methods in Fuchs et al. (2001), but with assigning of our own siderotype numbers.

d FePVD standard that was taken up in greatest amount. (% uptake compared to that of the homologous standard strain).

e Clinical specimen or from associated medical environment, Collection de la Faculté de Médecine de Lille, France (Meyer et al., 2007); freshwater pipe encrusted with MnO_2_, Germany (Schweisfurth, 1973); freshwater sediment, Green Bay of Lake Michigan, USA (Francis and Tebo, 2001); laboratory variant selected by Brandy Toner in the Garrison Sposito laboratory, University of California Berkeley, USA; marine fjord, oxic-anoxic interface, Saanich Inlet, Vancouver Island, BC, Canada (Emerson et al., 1982; Francis and Tebo, 2001; Romanenko et al., 2008); Metallogenium particles from Horsetooth Reservoir, Fort Collins, CO, USA (Francis and Tebo, 2001); pulpmill effluent, Grande Prairie, AB, Canada (Francis and Tebo, 2001); radiowaste leachate, low-level radioactive waste leachate, Brookhaven Natl. Lab., USA (Francis and Dodge, 1998); sediment, mine drainage, Pinal Creek, Globe, AZ, USA, downstream from a Cu mine (Fuller and Harvey, 2000; Francis and Tebo, 2001); soil, toluate deg, soil enrichment for degradation of toluate, Osaka, Japan (Nakazawa, 2002; Regenhardt et al., 2002).

f NT, not tested. Strain MG-1 did not grow at the standard conditions used for IEF analysis and ^59^Fe PVD uptake.

g NA, not applicable because that organism does not make PVD.

## Results

### *in silico* identification of the putative PVD synthesis operon in *p. putida* GB-1

**Figure 1:**
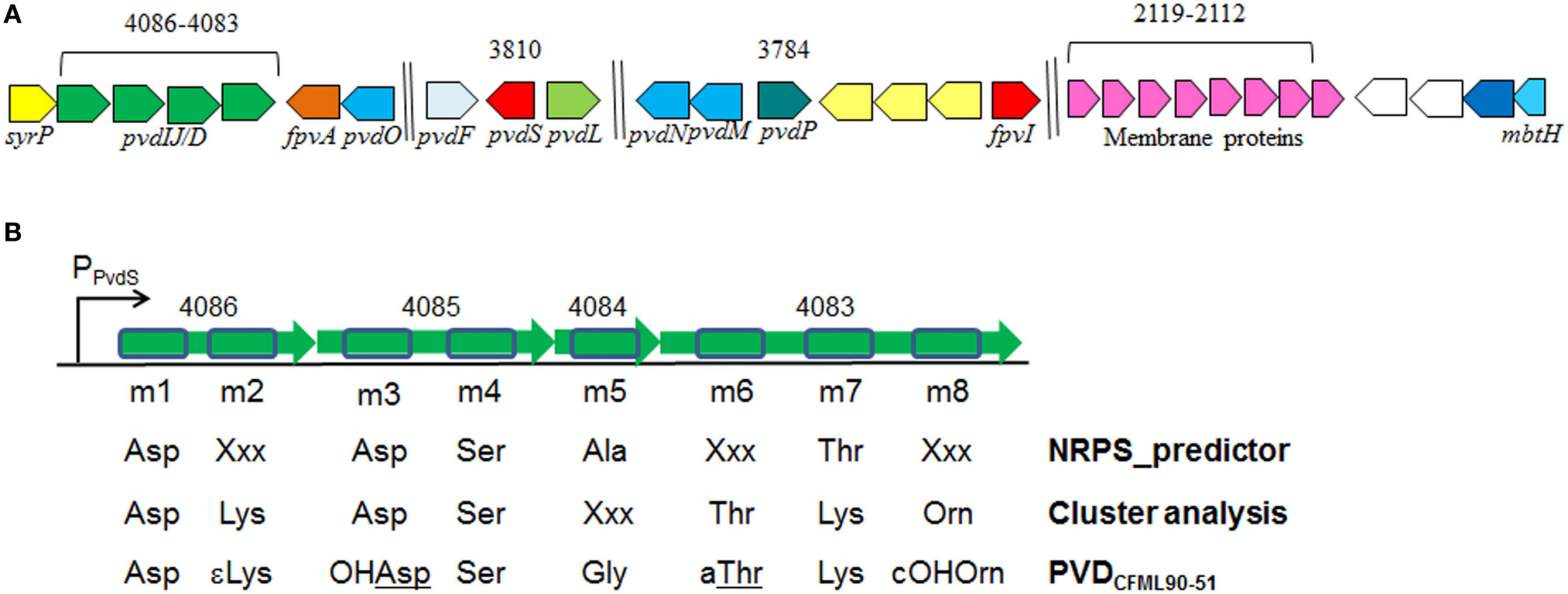
**(A)** Clusters of putative genes likely to be involved in PVD synthesis in *P. putida* GB-1. Identification is based on *in silico* comparison to known PVD-related genes, as described in the text. Genes are annotated according to Ravel and Cornelis (2003). Color schemes are based on Ravel and Cornelis (2003). Numbers on top are locus tags for *P. putida* GB-1 (PputGB1 numbers). Length is not to scale. **(B)** The 8 predicted adenylation domains found in the putative NRPSs (PputGB1_4083-4086) in *P. putida* GB-1, and the resulting peptides of each adenylation domain predicted using NRPSpredictor and cluster analysis in comparison with the reported peptide sequence of PVD_CFML90-51_. The NRPS genes are shown in green with the adenylation domains boxed in blue.

The genome of *P*. *putida* GB-1 encodes an NRPS operon comprised of the genes PputGB1_4086 through PputGB1_4083 (Figure 1) (Markowitz et al., 2008). These four genes are annotated as encoding NRPSs and have homology to the *P*. *aeruginosa* PAO1 PVD synthesis genes *pvdI*/*J* and *D*. Furthermore, downstream of this operon is a putative TonB-dependent siderophore receptor gene (PputGB1_4082). A gene encoding a homolog of PvdO (PputGB1_4081) which appears to have some role in PVD formation (Yeterian et al., 2010) was also found to be present (Figure 1A). Upstream of the first gene in the putative operon—PputGB1_4086—is a sequence with a perfect match to the PvdS sigma recognition site (TAAAT-N_16_-CGT) (Ochsner et al., 2002) (Figure 1B). The alternative sigma factor PvdS is an iron-responsive extracytoplasmic function (ECF) sigma (Leoni et al., 2000), suggesting that expression of the genes PputGB1_4086-4083 is regulated by iron concentration as would be expected for a PVD synthesis operon. A PvdS recognition site is located upstream of the *pvdI* (PA2402) and *pvdD* (PA2399) genes of *P*. *aeruginosa* PAO1 as well (Ochsner et al., 2002). PputGB1_3810, a homolog of *pvdS* (PA2424), was found along with PputGB1_3809, a homolog of *pvdL/psvA* (PA2424), a putative NRPS for chromophore synthesis (Mossialos et al., 2002) (Figure 1A). Also found in this operon along with NRPSs for PVD peptide backbone synthesis is a homolog of a gene encoding SyrP (PputGB1_4087) which is an Asp hydroxylase required for synthesis of syringomycin (Singh et al., 2008).

### Generation of an NRPS mutant

**Figure 2:**
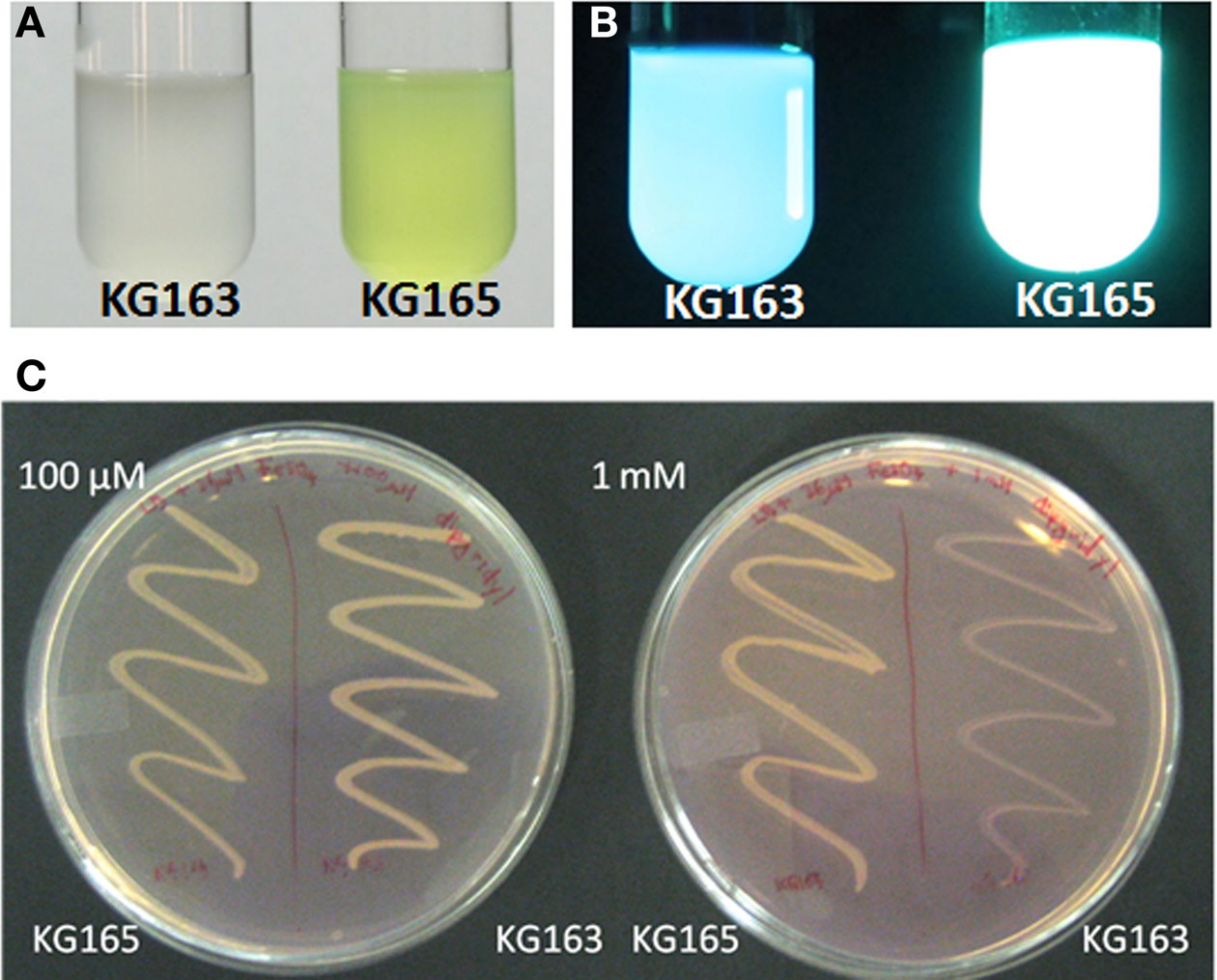
**KG163 (PVD synthesis mutant of *P. putida* GB-1) and KG165 (WT equivalent) grown in succinate medium and visualized under normal light (A) and UV (B)**. KG163 and 165 grown on LB supplemented with 36 μM FeSO_4_ and different amounts of 2'-2' dipyridyl **(C)**. Plate on the left has 100 μM and the right has 1 mM dipyridyl while the left side of each plate is the WT equivalent and on the right side of each plate is the NRPS mutant.

If the PputGB1_4086-4083 operon encodes the synthetic machinery of the PVD peptide in *P. putida* GB-1, disruption of this operon should lead to a loss of PVD synthesis, as has been shown to occur following mutation of the PVD peptide NRPS genes *pvdI* and *pvdD* in *P. aeruginosa* PAO1 (Merriman et al., 1995; Lehoux et al., 2000). Plasmid integration disruption was therefore performed on PputGB1_4083, which encodes a homolog to *pvdI* (PA2402 in *P. aeruginosa* PAO1). As would be expected, the plasmid integration mutant (KG163) did not fluoresce and lacked yellow-green pigments whereas a control strain in which the plasmid was integrated into the *att*Tn7 site (KG165) did, suggesting the NRPS mutant KG163 did not produce PVDs (Figures 2A,B).

KG163 was also grown in increasing amounts of the iron-chelator dipyridyl to verify the decreased ability to synthesize PVD, which is needed to compete for iron under these conditions. As shown in Figure 2C, the growth of the NRPS mutant is inhibited by increasing amounts of dipyridyl while growth of KG165 was not. This result supports the conclusion that the inability to acquire iron and not toxicity of dipyridyl was responsible for the growth defect of the NRPS mutant, KG163. Based on these findings, we conclude that PputGB1_4083 is required for PVD synthesis.

### *in silico* analyses of adenylation domains of NRPSs

**Figure 3:**
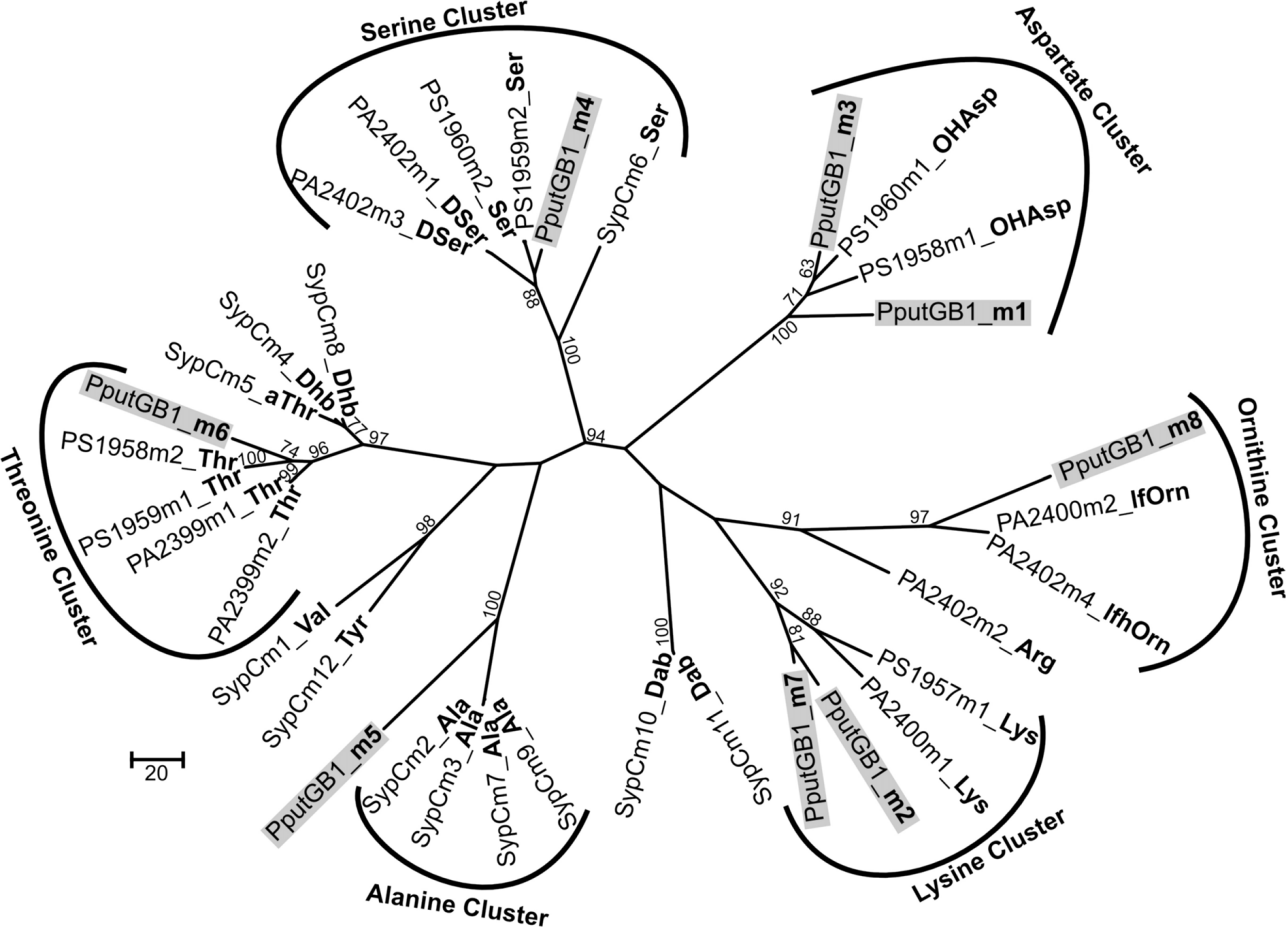
**Phylogenetic cluster analysis using MUSCLE performed for selected adenylation domains among pseudomonads along with 8 adenylation domains found in the genome of *P. putida* GB-1 in PputGB1_4086-4083**.

To determine the peptide sequence of PVD_GB−1_, *in silico* analyses were performed using the NRPSpredictor website (http://www-ab.informatik.uni-tuebingen.de/software/NRPSpredictor) (Rausch et al., 2005). *In silico* analyses predicted 8 adenylation modules within the 4 genes of the putative PVD peptide synthesis operon (PputGB1_4086-4083) (Figure 1B). Also, it was possible to generate a preliminary prediction of the sequence of amino acids in the peptide: Asp_1_-Xxx_2_-Asp_3_-Ser_4_-Ala_5_-Xxx_6_-Thr_7_-Xxx_8_ (where Xxx indicates amino acids that could not be predicted using NRPSpredictor). To further investigate the nature of the amino acids recognized by each of the adenylation domains in the putative PVD peptide synthesis operon of *P*. *putida* GB-1, we compared the amino acid sequence of each domain to that of other pseudomonad PVD synthesis proteins that produce PVDs with known structures. In this analysis, most of the adenylation modules from *P*. *putida* GB-1 fell into distinct clusters with known adenylation modules in other organisms (Figure 3). The cluster analysis confirmed the conclusions of NRPS predictor for modules m1 (Asp), m3 (Asp), and m4 (Ser), but suggested a Lys at m7 instead of the Thr chosen by NRPS predictor (Figure 1B). For the three cases in which NRPS predictor failed to select an amino acid, the identifications by cluster analysis were: m2 (Lys), m6 (Thr), and m8 (Orn) (Figure 1B). More precisely, module m6 clusters with other Thr-incorporating modules from five different pseudomonads, and it appears to be distinct from the allo-Thr incorporating modules of SypC, although these belong to the more distantly related organism *Pseudomonas syringae* pv. *syringae* B301D. Module m8 falls into a cluster with two *P*. *aeruginosa* PAO1 modules that incorporate N^5^-formyl-N^5^-hydroxyornithine (hfOrn) but is located on a long branch and therefore might direct incorporation of a different amino acid. However, the *P*. *putida* GB-1 genome encodes a homolog to PvdA (PputGB1_2120), which is an enzyme responsible for the modification of ornithine to OHOrn (Visca et al., 2007) and thus strain GB-1 is predicted to have the biochemical potential to generate OHOrn. Module m5 appears to cluster somewhat near several Ala-incorporating modules from *Pseudomonas syringae* pv. *syringae* B301D, but lies on a long branch outside of that cluster, which may reflect a lack of sufficiently homologous sequences to accurately root this branch on the tree.

### Chemical composition and siderotyping of PVD_GB−1_ and PVD_MnB1_

The PVDs of strains *P. putida* GB-1 and MnB1 were siderotyped in comparison to those of several isolates of known PVD structure (Table 2). Strains GB-1 and MnB1 were found to be in the same siderotype as *P. putida* CFML 90-51, which has been reported to have the following PVD peptide sequence: chromophore-Asp-Lys-OHAsp-Ser-Gly-aThr-Lys-cOHOrn (D-amino acids underlined) (Sultana et al., 2000). Since this sequence was strikingly similar to that predicted for PVD_GB−1_ by cluster analysis, it is included in Figure 1A for comparison. To determine whether this sequence also applied to strains GB-1 and MnB1, mass spectrophotometric (MS/MS) analysis was used to obtain molecular weights and also to identify the secondary MS/MS fragments of each PVD in partially purified preparations from the *P. putida* strains GB-1 and MnB1, in comparison to parallel preparations from *P. putida* CFML 90-51 (Table 3). The PVDs of all three organisms shared a major MS peak at the monoisotopic *m/z* of 1250–1251 Da (Table 3). This value, as well as the weights of secondary MS/MS fragmentation products, corresponded to the peptide sequence given above. Our data agreed with the previous report for PVD_CFML90−51_ (Sultana et al., 2000), except that the variable acyl side chain in our samples from strain GB-1 was malic acid amide (mala) instead of malic acid (mal) and the acyl side chain in our CFML 90-51 preparation was ~50% mala and ~50% mal, in contrast to the previous report in which only mal was found with CFML 90-51 (Sultana et al., 2000). Interestingly, MS/MS samples from all three strains, CFML 90-51, MnB1, and GB-1, also contained a major monoisotopic peak at *m/z* 1161 Da, which was identified to contain the same PVD peptide as in the 1250 Da peak, but with the PVD chromophore replaced by an azotobactin chromophore, a structure that is frequently co-produced with pyoverdine (Fuchs et al., 2001). Table 2 lists the MS apparent relative abundance of the peaks at m/z of 1250.4 and 1161.4 Da, expressed as a percentage of the total peak area of PVD-type siderophores produced by each strain. The samples also showed minor amounts of sulfonated PVD at 1333.5–1335.5 Da (2 and 6% of the total peak areas, respectively), and two minor peaks at 1232.5 and 1144 Da, which appear to represent water-loss reactions involving β elimination at various hydroxyl groups of the peptide, with the formation of double bonds conjugated to a carbonyl. Material from each peak at *m/z* 1250, 1161, and 1333 Da was further fragmented and analyzed by MS/MS. Since these secondary fragments from the three tested siderotype n° 1 strains were indistinguishable (data not shown), we conclude that these strains produced the same general set of PVD-type siderophores including sulfonated and non-sulfonated PVD and azotobactin.

**Table 3:** **Mass spectrometric (MS/MS) analysis of siderophores from several siderotype n° 1 strains of *Pseudomonas putida***.

***P. putida strain***	**Pyoverdine m/z (isotope cluster area, %)^a^**	**Azotobactin m/z (isotope cluster area, %)^a^**
CFML 90-51	1251.57 (53.6%)	1161.52 (26.8%)
GB-1	1250.57 (30.7%)	1161.53 (53.9%)
MnB1	1250.34	1161.34

a The mass spectrometric (MS) isotope cluster area is the sum of the peak areas of all isotopes associated with each monoisotopic m/z measurement, expressed as a percentage of all PVD-type molecules made by that strain. The peptide sequence of each siderophore was determined by analysis of the second sequential set of MS/MS peaks and was, in all cases: chromophore-Asp-Lys-OHAsp-Ser-Gly-aThr-Lys-cOHOrn. The small differences in weights and slight variations in malonate substitution are described in the text.

### Comparison of mn-oxidizing and mn-non-oxidizing strains

*Pseudomonas* isolates from diverse habitats and taxonomic groupings were characterized with respect to MnO_2_ formation and siderophore production (Table 2). The strains comprised six 16S rRNA groupings: *P. putida, P. fluorescens, P. marincola, P. fluorescens-syringae* group, *P. mendocina-resinovorans* group, and *P. stutzeri* group (Table 2). When iron starved, most strains were positive in the chrome azurol S (CAS) assay (Table 2), a standard method that detects the production of most types of siderophores (Schwyn and Neilands, 1987). The majority also formed PVD, as indicated by their release of green fluorescent compounds into the medium under iron-limiting, but not iron-replete, conditions (Table 2). The presence of PVD in culture fluids was confirmed by examination of fluorescence and absorbance spectra at pH 5, 6, and 8, both in the absence and presence of iron (Parker et al., 2004, 2007); in all positive cases the spectra were those expected for PVD (data not shown because they were so standard).

When the above strains were siderotyped based on the isoelectric focusing (IEF) patterns of their PVDs (Fuchs et al., 2001; Meyer, 2007), seven *P. putida* isolates showed identical IEF patterns (Table 2); these strains included two Mn(II) oxidizers (strains GB-1 and MnB1) and five isolates that did not detectably oxidize Mn(II) (the five CFML strains in Table 2). These seven strains also incorporated ^59^Fe-PVD from the reference organism CFML 90-51 as efficiently as their own ^59^Fe-PVD (Table 2), but did not internalize 24 other ^59^Fe-PVD standards of other siderotypes (see Methods). Based on our data and previous characterizations of the five CFML strains (Meyer et al., 2007), we conclude that these 7 isolates are of the same siderotype, here designated siderotype number 1 (n° 1), which is equivalent to n° 30 in Fuchs et al. (2001), siderovar n° 15 in Meyer et al. (2007), or n° 32 in Meyer et al. (2008).

The other tested strains were assigned to three new siderotypes (Table 2) that did not correspond to any grouping in Fuchs et al. (2001). *P. putida* KT2440 is here designated as siderotype n° 2, for which a PVD structure has been deduced from genome sequence (Ravel and Cornelis, 2003; Meyer et al., 2007). Previously, this siderotype was also assigned as siderovar n° 25 (Meyer et al., 2007) or as type 3 (Matthijs et al., 2009).

Siderotype n° 3, *P. fluorescens* ATCC 55241, did not take up any tested ^59^Fe-PVD standard and appears to have an as yet uncharacterized PVD (Table 2). The siderotype n° 4 strains were *P fluorescens-syringae* group Mn^(II)^ oxidizers ISO6 and PCP1 which partially took up ^59^Fe-PVD of two reference strains, *P. fluorescens* SB8.3 and *Pseudomonas* sp. D47 (Table 2). PVD of *Pseudomonas* sp. D47 was previously classified as n° 29 and *P. fluorescens* sp. SB8.3 (=Ps4a) as n° 7 (Meyer et al., 2008). Although we have not determined the PVD structures of siderotypes n° 3 and n° 4, it is clear that they are not the same as those of siderotypes n° 1 or n° 2.

## Discussion

*P. putida* strains GB-1 and MnB1 are Mn(II)-oxidizing bacteria that produce pyoverdines (PVDs), siderophores that affect iron uptake but may also substantially influence the metabolism of other metals, including Mn. To facilitate future studies of the potentially multi-faceted roles such as inhibiting Mn(II) oxidation played by PVD in the oxidation of Mn by pseudomonads, we have examined both the genetics of PVD production in strain GB-1 and the composition of the pyoverdines produced by *P. putida* GB-1, as well as several other Mn(II)-oxidizing strains.

Within the genome sequence of *P. putida* GB-1, a putative non-ribosomal peptide synthase (NRPS) operon capable of being involved in the synthesis of the PVD_GB−1_ peptide was identified: PputGB1_4086-4083. Disruption of PputGB1_4086-4083 resulted in a defect in PVD synthesis similar to that reported in other *Pseudomonas* strains with mutations in comparable NRPSs (Merriman et al., 1995; Lehoux et al., 2000). Furthermore, the peptide sequence based on i*n silico* analyses of the adenylation domains of NRPS PputGB1_4086-4083 was consistent with the actual peptide sequence determined from MS/MS of PVD_GB−1_ samples. Therefore, it can be safely concluded that PputGB1_4086-4083 is the NRPS that governs PVD peptide synthesis in *P. putida* GB-1. *In silico* examination of the *P. putida* GB-1 genome also identified candidates (Figure 1) for many of the other genes needed for the synthesis, modification, and expression of PVD_GB−1_, including a NRPS for chromophore synthesis (PputGB1_3809), a TonB-dependent siderophore receptor gene (PputGB1_4082), an Asp hydroxylase (PputGB1_4087), an Orn hydroxylase (PputGB1_2120), and an iron-responsive ECF alternative sigma factor (PputGB1_3810) with recognition sites found in the promoters upstream of most of the above genes.

Based on siderotyping followed by MS/MS analysis (Tables 2, 3), we have determined the peptide sequences of PVD_GB−1_ and PVD_MnB1_ to be: chromophore-Asp-Lys-OHAsp-Ser-Gly-aThr-Lys-cOHOrn, identical to that reported (Sultana et al., 2000) and confirmed here (Table 3) for PVD_CFML90−51_. The OHAsp and the aThr in PVD_CFML90−51_ are known to be D isomers (Sultana et al., 2000). PVD_GB−1_ and PVD_MnB1_ probably also contain these two D amino acids because the degree of uptake of ^59^Fe-labeled PVD_CFML90−51_ by strains GB-1, MnB1, and CFML 90-51 was similar (Table 2), suggesting that the cellular uptake receptors of strains GB-1 and MnB1 did not detect a steric difference between PVD_CFML90−51_ and the endogenous PVD of each strain. However, the presence of D amino acids in PVD_GB−1_ and PVD_MnB1_ has not been directly tested.

The peptide sequence above suggests a metal-binding pocket formed by three moieties: (1) the catecholate of the chromophore, (2) the cyclic hydroxamate from cOHOrn, and (3) the α-OH-carboxylate from OHAsp. It is not yet clear how this structure, including the presence of a (OH)carboxylate donor group, leads to the higher thermodymamic stability constants for Mn(III), as compared to Fe(III), reported for these siderophores at physiological and alkaline pH (Parker et al., 2004; Harrington et al., 2012). However, an influence of OHcarboxylate on the preferential binding of Mn(III) has been recently suggested by an investigation showing that siderophores containing solely hydroxamates (e.g., desferrioxamine B, DFOB) or solely catecholates (e.g., protochelin) bind Fe(III) more strongly than Mn(III), whereas rhizoferrin, which complexes metals via a mixture of carboxylate and (OH)carboxylate groups, binds Mn(III) more strongly than Fe(III) (Harrington et al., 2012), as do several aminocarboxylate ligands (Hamm and Suwyn, 1967; Ahrland et al., 1990; Martell and Smith, 2003). Based on K-edge EXAFS comparisons of various Fe(III)- and Mn(III)-siderophore complexes in solution, Harrington et al. (2012) proposed that the greater flexibility of carboxylates *vis-à-vis* hydroxamates or catecholates allows the former to accommodate the Jahn-Teller-distorted coordination that is characteristic of Mn(III) but not Fe(III). This result suggests that (OH)carboxylates within siderophores, perhaps including the siderotype n° 1 PVDs studied here, may affect the preferential binding of Mn(III). However, the situation for pyoverdines probably also involves additional factors, because two mixed-moiety pyoverdines (PVD_CFML90−51_ and PVD_Pa1_) both preferentially bound Mn(III) and both seemed to accommodate Jahn-Teller distortion, even though PVD_CFML90−51_ contains a (OH)carboxylate but PVD_Pa1_ does not (Harrington et al., 2012). Perhaps these PVDs gain flexibility from their mixed donor groups, their polypeptide structure, or some other factor.

MS/MS analysis of the PVDs from *P. putida* strains GB-1 and MnB1 also indicated that each strain produced a set of three PVD-type siderophores sharing the same peptide tail but with differently modified chromophores: “classical” PVD, sulfonated PVD, and azotoactin (Table 3). Since all three are strongly fluorescent and since fluorescence was undetectable in the mutant KG163 (Figure 2B), it is likely that the peptide tail of all three PVD types in strain GB-1 is synthesized through the same NRPS operon, PputGB1_4083-4086, which makes sense since the peptides of all three PVD types showed identical MSMS fragmentation patterns. However, subsequent modifications could be subject to differing regulatory or catalytic pathways. It is currently unknown whether these three differing PVD types affect Mn metabolism or the complexation of Mn *vis á vis* Fe similarly or differently.

Azotobactin and PVD are both known to complex various metal cations (Braud et al., 2009; Wichard et al., 2009). However, azotobactin can also bind oxyanions such as molybdate and vanadate (Wichard et al., 2009). In contrast, the predominant PVD of *P. aeruginosa* PAO1 is not able to form complexes with vanadate, whereas the other main siderophore of strain PAO1, pyochelin, can (Baysse et al., 2000). This observation is consistent with other reports that PVDs do not play an important role with oxyanions (Wichard et al., 2009). Therefore, one function of azotobactin in *P. putida* GB-1 and related strains might be to complex oxyanions for uptake or detoxification, as was suggested for *Azotobacter vinelandii* (Wichard et al., 2009). Alternatively, sulfonated PVDs and azotobactin could be precursors or byproducts of PVD synthesis (Fuchs et al., 2001; Baysse et al., 2002). Further studies need to be performed to elucidate the respective roles of the multiple siderophores of these organisms, especially with regard to manganese oxidation.

Since the ability to oxidize Mn(II) occurs very commonly, but nonetheless sporadically, among a wide variety of *Pseudomonas* species (Francis and Tebo, 2001), it was no surprise that multiple PVD siderotypes were identified among the phylogenetically-diverse Mn(II)-oxidizing pseudomonads tested here (Table 2). Within *P. putida, two* siderotypes were identified (Table 2): n° 1 including strains CFML 90-51 and GB-1 and n° 2 consisting of strain KT2440. Although not included in this research, *P. putida* ATCC 12663 is also capable of oxidizing Mn(II), is closely related to *P. putida* GB-1 based on 16S data (Francis and Tebo, 2001), but produces a PVD that has been previously shown to be different from PVD_CFML90−51_ (Meyer et al., 2007). Since PVD_CFML90−51_ and PVD_GB−1_ are indistinguishable by MS/MS (Table 3) and siderotyping (Table 2), PVD_GB−1_ and PVD_ATCC12663_ cannot be the same. Therefore, even among the Mn(II)-oxidizing *P. putida* at least three differing PVDs exist: PVD_GB−1_, PVD_KT2440_, and PVD_ATCC12663_. This situation is in agreement with the conclusion of Meyer et al. (2007) that *P. putida* as currently defined is heterogeneous with respect to siderotype. It is also notable that the siderotype of a Mn oxidizer can be the same as that of a strain that does not oxidize Mn(II), as for *P. putida* GB-1 and CFML 90-51 (Table 2).

In summary, this study has combined *in silico*, genetic and chemical (siderotyping and MS/MS) approaches to explore the synthesis and nature of the suite of related PVDs (“classic” PVD, azotobactin, and sulfonated PVD) that were produced by the model Mn(II)-oxidizing organism *Pseudomonas putida* GB-1 at our growth conditions. *In silico* analysis indicated that position PputGB1_4083-4086 of the GB-1 genome contained NRPSs that could synthesize a peptide chain consistent with the PVD_GB−1_ peptide determined by MS/MS (chromophore-Asp-Lys-OHAsp-Ser-Gly-aThr-Lys-cOHOrn). Furthermore, mutation at PputGB1_4083 prevented PVD synthesis. A diverse selection of Mn-oxidizing *Pseudomonas* species were found to comprise at least three distinct PVD siderotypes, indicating differences in PVD structure and PVD uptake specificity that can be exploited in future studies concerning the ways that various PVDs can influence Mn metabolism, especially Mn(II) oxidation, in pseudomonads and other bacteria.

### Conflict of interest statement

The authors declare that the research was conducted in the absence of any commercial or financial relationships that could be construed as a potential conflict of interest.
